# Long non-coding RNA SPRY4-IT1 pormotes colorectal cancer metastasis by regulate epithelial-mesenchymal transition

**DOI:** 10.18632/oncotarget.10407

**Published:** 2016-07-06

**Authors:** Fei Shen, Wen-Song Cai, Zhe Feng, Ji-wei Chen, Jian-hua Feng, Qi-cai Liu, Yong-ping Fang, Kun-ping Li, Huan-qing Xiao, Jie Cao, Bo Xu

**Affiliations:** ^1^ Department of General Surgery, Guangzhou First People's Hospital, Guangzhou Medical University, Guangzhou, P.R. China; ^2^ Experimental Medical Research Center, Guangzhou Medical University, Guangzhou, P.R. China; ^3^ Department of General Surgery, Huizhou First People's Hospital, Huizhou, P.R. China

**Keywords:** SPRY4-IT1, LncRNA, CRC, metastasis, EMT

## Abstract

Colorectal cancer (CRC) remains one of the most common cancers worldwide. Increasing evidence indicates that SPRY4 intronic transcript 1 (SPRY4-IT1) regulate cell growth, differentiation, apoptosis, and cancer progression. However, the expression and function of SPRY4-IT1 in the progression of CRC remains largely unknown. Here, we reported that SPRY4-IT1 was upregulated in CRC. Increased SPRY4-IT1 expression in CRC was associated with larger tumor size and higher clinical stage. *In vitro* experiments revealed that SPRY4-IT1 knockdown significantly inhibited CRC cell proliferation by causing G1 arrest and promoting apoptosis, whereas SPRY4-IT1 overexpression promoted cell proliferation. Further functional assays indicated that SPRY4-IT1 overexpression significantly promoted cell migration and invasion by regulate the epithelial-mesenchymal transition (EMT). Taken together, our study demonstrates that SPRY4-IT1 could act as a functional oncogene in CRC, as well as a potential therapeutic target to inhibit CRC metastasis.

## INTRODUCTION

Colorectal cancer (CRC) is associated with a high mortality rate due to its rapid progression and advanced tumor presentation [1–3]. Although preoperative treatment with chemoradiotherapy in combination with conventional surgery improves overall survival, metastasis of CRC is correlated closely with poor prognosis [4–5]. Therefore, it is urgent to identify new therapeutic strategies.

Long non-coding RNAs (lncRNAs, > 200 nucleotides in length) are important new members of the family of ncRNAs with limited or no protein-coding capacity [7–8]. Accumulating evidence has shown that lncRNAs are emerging as key molecules in human malignancies [9–11]. Recently, increasing evidence has shown that SPRY4 intronic transcript 1 (SPRY4-IT1), belonging to a group of intron-retained lncRNAs, was shown to be upregulated in various cancer [12–13]. SPRY4-IT1 was reported to have high expression in melanoma, renal cancer, and breast cancer. Knockdown of SPRY4-IT1 inhibited cell proliferation, migration, and invasion, and induced apoptosis of cancer cells. However, its expression pattern, clinical performance and functional roles in CRC had not been addressed. In this study, we design the experiments to investigate the involvement of the lncRNA SPRY4-IT1 in the tumorigenesis and cancer progression of CRC.

## RESULTS

### SPRY4-IT1 is highly expressed in CRC samples and cell lines

**Figure 1:**
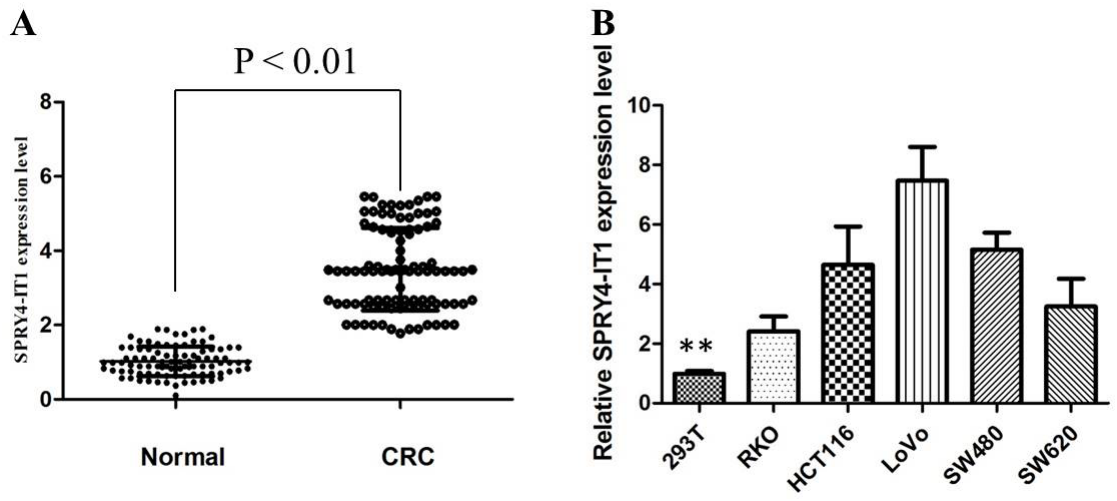
The SPRY4-IT1 expression levels in CRC tissues and cell lines **A**. SPRY4-IT1 was detected in CRC tissues and adjacent noncancerous tissues by qRT-PCR. **B**. qRT-PCR showing expression level of SPRY4-IT1 in CRC cell lines.

Firstly, we analyzed the expression of SPRY4-IT1 in 96 pair of CRC tumor and non-cancerous tissues by qRT-PCR. The relative expression of SPRY4-IT1 in CRC tissues compared with normal tissues is shown in Figure 1A. The expression of SPRY4-IT1 was significantly increased in 71% of CRC tissue samples (68/96), compared with adjacent non-tumor tissues, indicating that SPRY4-IT1 might play an oncogenic role in CRC. Furthermore, a correlation analysis of SPRY4-IT1 expression with clinicopathological parameters revealed that the SPRY4-IT1 expression level was predominantly increased in late-stage tumor tissues and positively correlated with tumor size (*P* < 0.01).

Then, we evaluated the expression of SPRY4-IT1 in a panel of human CRC cell lines by qRT-PCR. The expression of SPRY4-IT1 was observed to be higher in all five CRC cell lines compared with the 293T cell line. As shown in Figure 1B, the result of qRT-PCR revealed that LoVo and SW480 cells showed higher expression of SPRY4-IT1; however, RKO showed lower expression of SPRY4-IT1. Thus, we used LoVo, SW480, and RKO cells as a model to investigate the function of SPRY4-IT1 on cell proliferation, apoptosis, migration and invasion.

### SPRY4-IT1 regulates cell growth and the cell cycle in CRC cells

**Figure 2:**
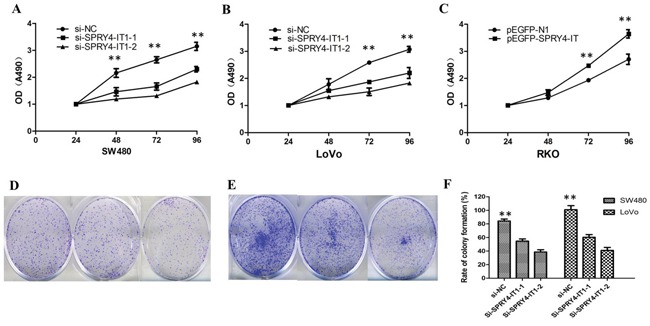
**A**. MTT assay showing knockdown of SPRY4-IT1 inhibited cell proliferation of SW480 cells. **B**. MTT assay showing knockdown of SPRY4-IT1 inhibited cell proliferation of LoVo cells. **C**. MTT assay showing overexpression of SPRY4-IT1 increased proliferation of RKO cells. **D**. Colony-formation assays showed that silencing of SPRY4-IT1 significantly increased the colony-forming ability of SW480 cells. **E**. Colony-formation assays showed that silencing of SPRY4-IT1 significantly increased the colony-forming ability of LoVo cells. **F**. The quantification of rate of colony-formation is presented.

**Figure 3:**
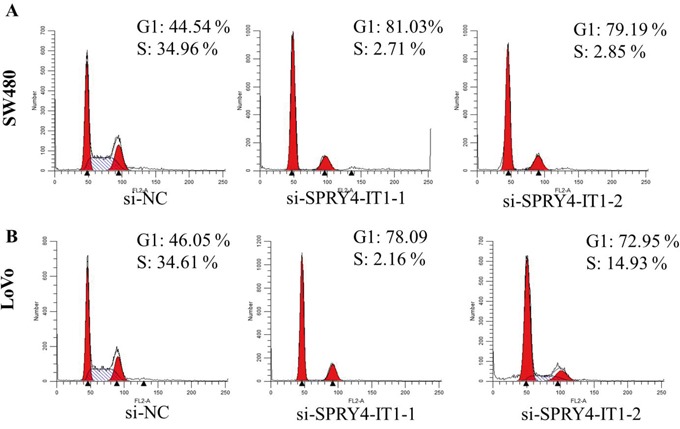
**A**. SW480 cells transfected with si- SPRY4-IT1 all had cell-cycle arrest at the G1-G0 phase compared with cells transfected with si-NC. **B**. LoVo cells transfected with si-SPRY4-IT1 had cell-cycle arrest at the G1-G0 phase compared with cells transfected with si-NC.

To further examine whether SPRY4-IT1 is involved in CRC progression, *in vitro* functional analyses were performed. Knockdown of SPRY4-IT1 by RNAi significantly decreased proliferation of SW480 and LoVo cells (Figure 2A and 2B) and overexpression of SPRY4-IT1 increased proliferation of RKO cells (Figure 2C). Consistently, colony formation assay showed that knockdown of SPRY4-IT1 significantly reduced the growth of LoVo and SW480 cells, suggesting that SPRY4-IT1 may act as an oncogene involved in the promotion of CRC cell proliferation (Figure 2D-2F). To further investigate the growth inhibition observed following SPRY4-IT1 knockdown, we compared the cell-cycle profiles of SPRY4-IT1 knockdown cells and controls by flow cytometry. Suppression of SPRY4-IT1 led to a decrease in the number of cells in the S-phase and an increase in the percentage of cells in the G0/G1 phase (Figure 3A and 3B).

### Downregulation of SPRY4-IT1 causes apoptosis in CRC cells

**Figure 4:**
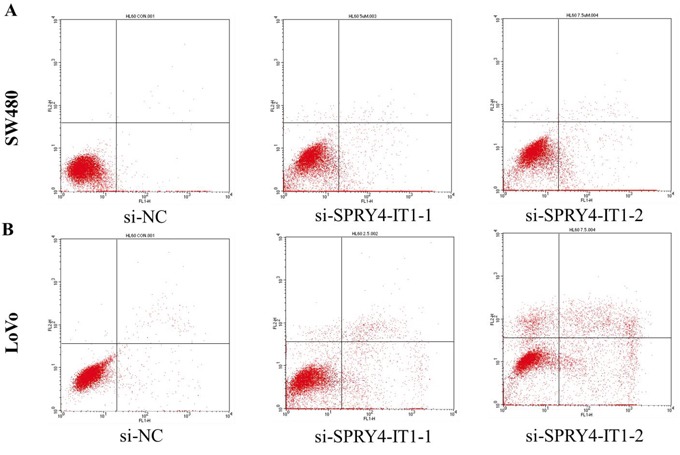
**A**. The proportion of apoptotic cells following SPRY4-IT1 siRNA treatment was increased in SW480 cells. **B**. The proportion of apoptotic cells following SPRY4-IT1 siRNA treatment was increased in LoVo cells.

In addition, we performed flow cytometry to examine whether CRC cell proliferation was induced by cell apoptosis. The results revealed that knockdown of SPRY4-IT1 could obviously induce cell apoptosis, the proportion of apoptotic cells following SPRY4-IT1 siRNA treatment was increased in LoVo and SW480 cells (Figure 4A and 4B), suggesting that SPRY4-IT1-mediated promotion of CRC cell proliferation seems to be mediated by regulation of apoptosis.

### SPRY4-IT1 regulates CRC cell invasion and epithelial-mesenchymal transition (EMT)

**Figure 5:**
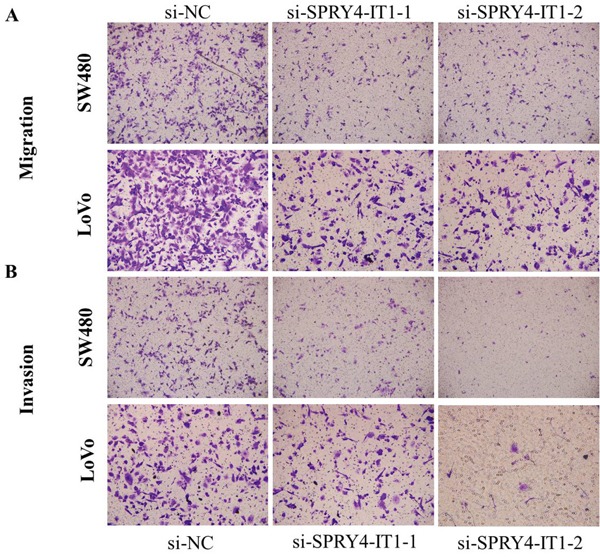
**A**. Inhibition of Migration and Invasion of SW480 cells by SPRY4-IT1 siRNA. **B**. Inhibition of Migration and Invasion of LoVo cells by SPRY4-IT1 siRNA.

**Figure 6:**
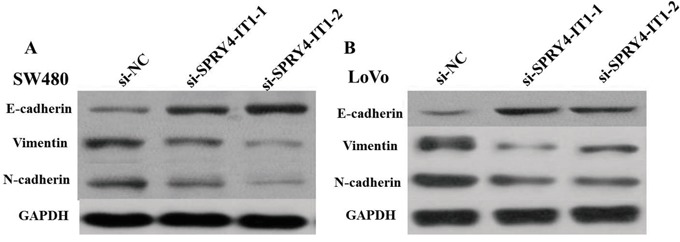
**A**. Knockdown of SPRY4-IT1 reverses EMT in SW480 cells. **B**. Knockdown of SPRY4-IT1 reverses EMT in LoVo cells.

Cell motility, invasiveness and colony formation ability strongly correlate with cancer metastasis. Therefore, we next investigated the effect of SPRY4-IT1 on these characteristics of CRC cells. We found that SPRY4-IT1 knockdown dramatically decreased their migration and invasion capabilities, respectively. Quantification of invading cells revealed a significant decrease in the number of invading cells for both cell lines after SPRY4-IT1 knockdown (Figure 5A and 5B). Conversely, the migration activity of SPRY4-IT1-overexpressing cells was significantly increased. To measure the effect of silencing SPRY4-IT1 expression on EMT of CRC cells, western blot was performed to examine the expression of EMT-related markers in LoVo and SW480 cells after transfection with si-SPRY4-IT1. As expected, SPRY4-IT1 knockdown remarkably increased the expression of E-cadherin and meanwhile greatly decreased the expression of N-cadherin and Vimentin in LoVo and SW480 cells (Figure 6A and 6B), indicating that downregulation of SPRY4-IT1 obviously blocked the EMT process.

## DISCUSSION

CRC is one of the most common malignancies, which ranks the third in the cancer morbidity and the second in the cancer mortality worldwide, with over 1.2 million new cases each year. The lncRNAs are important new members of the ncRNA family, which are greater than 200 nt in length and can be transcribed by RNA polymerase II (RNA pol II). Recent, evidence has indicated that many lncRNAs have been shown to aberrantly express in human cancers and involved in carcinogenesis [14–16]. In CRC, expression of lncRNAs has only been analysed in a few studies [17–20]. Besides, the functions and molecular mechanisms of most lncRNAs have not been well characterized. Therefore, the identification of CRC-associated lncRNAs and investigation of their molecular and biological functions are important to provide new insights into the diagnosis and treatment of CRC.

SPRY4-IT1 (GenBank accession ID AK024556), is derived from the intronic region of the Sprouty4 gene, and is predicted to contain several long hairpins in its secondary structure [21]. Elevated expression of SPRY4-IT1 was associated with poor prognosis of various tumors [22–23]. SPRY4-IT1 was previously reported to be upregulated in melanoma cells, and knockdown of its expression led to cell growth arrest, invasion inhibition, and elevated rates of apoptosis. However, the influence of SPRY4-IT1 on CRC has been not reported. In this study, we explored the expression pattern of SPRY4-IT1 in CRC tissues and cell lines, and investigated the effects of SPRY4-IT1 expression on CRC cell phenotypes *in vitro*. First, we measured the expression level of SPRY4-IT1 in clinical CRC tissues by qRT-PCR. We confirmed that SPRY4-IT1 is extremely upregulated in most of CRC tissues, compared with normal tissues, indicating that SPRY4-IT1 may primarily participate in CRC. Besides, SPRY4-IT1 was obviously upregulated in a panel of CRC cell lines. The qRT-PCR results revealed that LoVo and SW480 cells showed higher expression of SPRY4-IT1, and RKO showed lower expression of SPRY4-IT1.

We then determined whether SPRY4-IT1 expression influences tumor-like characteristics such as proliferation and apoptosis [24]. Our results showed that knockdown of SPRY4-IT1 by siRNA in LoVo and SW480 cells was shown to significantly inhibit CRC cell growth and colony formation, and increase cell apoptosis, whereas the ectopic expression of SPRY4-IT1 significantly enhances cell proliferation in RKO cell lines. Moreover, suppression of SPRY4-IT1 led to a decrease in the number of cells in the S-phase and an increase in the percentage of cells in the G0/G1 phase, suggeating that SPRY4-IT1 may affect CRC progression by affecting cell proliferation and apoptosis.

Although SPRY4-IT1 is involved in metastasis of different cancers, little is known about the underlying molecular mechanism. EMT is an essential process for tumor invasion and metastasis [25]. In the present study, we identified that knockdown of SPRY4-IT1 expression can suppress migratory and invasive phenotype of LOVO and SW480 cells. In accordance with this, knockdown of SPRY4-IT1 expression increased the expression levels of E-cadherin and meanwhile greatly decreased the expression of Vimentin, indicating that SPRY4-IT1 affects CRC metastasis partly via the EMT.

In summary, we have shown that SPRY4-IT1 was upregulated in CRC tissues and could act as a functional oncogene in CRC cell lines. Thus, these results indicate that SPRY4-IT1 may become a novel promising candidate for the therapy for CRC.

## MATERIALS AND METHODS

### Human tissue

In the study, a total of 96 paired samples of CRC and adjacent non-tumor tissues (more than 5 cm away from the tumor) were obtained from CRC patients undergoing surgical procedures at Guangzhou First People's Hospital of Guangzhou Medical University. Both tumors and noncancerous tissues were subjected to histological analysis for diagnostic confirmation. All samples were immersed immediately in RNAlater solution (Ambion, Austin, Texas) overnight, then stored at -80°C in order to avoid degradation of RNA. Prior to the use of these clinical materials for research purposes, written consents from all patients and approval of Guangzhou Medical University Ethic Review Committees were obtained.

### Cell lines

Five CRC cell lines (HCT116, LoVo, RKO, SW620, and SW480) and the 293T cell line were purchased from the Institute of Biochemistry and Cell Biology of the Chinese Academy of Sciences (Shanghai, China). Cells were cultured in RPMI 1640 or DMEM (Gibco, Grand Island, NY, USA) medium supplemented with 10% FBS, 100 U/ml penicillin, and 100 mg/ml streptomycin (Gibco) in humidified air at 37°C with 5% CO_2_.

### RNA isolation and quantitative real-time reverse transcription-PCR (qRT-PCR)

Total RNA was extracted from clinical samples and cell lines by TRIZOL reagent (Life Technologies, Foster City, CA, USA) and treated with DNase I (Invitrogen, Carlsbad, CA, USA) to eliminate potential DNA contamination. The GeneAmp RNA PCR kit (Life Technologies) was used to reverse-transcribe RNA to complementary DNA for the gene expression analysis. For qRTPCR, three replicates of each sample were amplified in a 20-μL reaction mixture containing SYBR Green reaction mix (Qiagen, Germany) and 0.5 mM of primer, and analyzed using a Roche Light-Cycler (Roche, Basel, Switzerland). The sequence of the primers were as following: SPRY4-IT1 (Forward: 5’-AGCCACATAAATTCAGCAGA-3’, Reverse: 5’-C GATGTAGTAGGATTCCTTTCA-3’) and GAPDH (Forward: 5’-GACTCATGACCACAGTCCATGC-3’, Reverse: 5’-AGAGGCAGGGATGATGTTCTG-3’). An ABI 7500 was used to carry out the qPCR and data collections.

### Small interfering RNA and plasmids DNA transfections

The cells were transiently transfected with si-RNAs after being sowed into the 6-well plates overnight. A scrambled negative control, a plasmid overexpressing SPRY4-IT1, and an empty vector, were cultured as well using the Lipofectamine 2000 transfection reagent (Invitrogen, Carlsbad, CA) and FuGENE^®^ HD Transfection Reagent (Roche, Germany) according to the manufacturer's instructions, respectively. Forty eight hours after transfection, the cells were harvested to detect the overexpression or knockout efficiency via qRT-PCR. For RNAi-mediated knockdown of SPRY4-IT1, two different Stealth siRNAs against SPRY4-IT1 were provided by Invitrogen. The target sequences for the si-SPRY4-IT1 included: si-SPRY4-IT1-1 (CCCAGAATGTTGACAGCTGCCTCTT) and si-SPRY4-IT1-2 (GCTTTCTGATTCCAAGGCCTATTAA).

### Cell proliferation assay

Cell proliferation was analyzed using MTT assay. Briefly, ~1×103 cells were seeded into a 96-well plate and incubated for 1, 2, 3, and 4 days. At the indicated time point, 20 μL of MTT (5 mg/mL) (Sigma-Aldrich) was added into each well and incubated for another 4 hours. Then the supernatants were removed and 150 μL of DMSO (Sigma-Aldrich) was added to terminate the reaction. The absorbance value (optical density [OD]) was measured at 490 nm on a microplate reader (Molecular Devices, Sunnyvale, CA, USA).

### Colony formation assays

For the colony formation assay, 1,000 cells were plated into each well of a six-well plate and were maintained in media containing 10 % FBS to allow colony formation, with the medium being repalaced every four days. After two weeks, colonies were fixed with methanol and stained with 0.1 % crystal violet (Sigma) in PBS for 15 min. The visible colonies were manually counted. Triplicate wells were measured for each treatment group.

### Determination of cell apoptosis by flow cytometry

After transfection, apoptosis was detected using the Annexin V-FITC Apoptosis Detection Kit. Cells were detached by trypsinization and washed three times in PBS, centrifuged at 1,000×g for 5min and resuspended in 195 μl Annexin V-FITC binding buffer. 5μl Annexin V-FITC was added and mixed. Then, the cells were stained in the dark for 10min at room temperature. After that, cells were centrifuged at 1,000×g for 5min and resuspended in 190 μl of Annexin V-FITC binding buffer. Last, 10 μl propidium iodide staining solution was added and mixed. The cells were kept on ice in the dark and immediately subjected to flow cytometry analysis. The data were analyzed using the Cell Quest software. The experiment was repeated three times.

### Migration and invasion assay

Cell migration and invasion assays were performed using transwell chambers (8 μm pore size; BD Biosciences). For the migration assay, approximately 1×10^5^ cells in serum-free media were seeded into the upper chambers after transfection for 48h. The lower chamber contained medium with 20% fetal bovine serum as a chemoattractant. Following a 24-hour incubation, the cells located on the lower surface of the chamber were stained and counted using a microscope (Olympus Corp., Tokyo, Japan). The invasion assay protocol was similar to the migration assay except that the upper chambers were first covered with Matrigel.

### Western blotting assay

Cells were lysed in the cell lysates (Thermo) supplemented with protease inhibitors PMSF and Cocktail (Roche). Proteins were separated in 8% sodium dodecyl sulfate-polyacrylamide gel electrophoresis and transferred to nitrocellulose NC membranes (0.22 mm, Whatman). Membranes were blocked with blocking buffer (Li-COR), sequentially incubated in primary antibodies and secondary antibody. The primary antibodies included anti-E-cadherin, anti-N-cadherin, anti-Vimentin, and anti-human GAPDH (Santa Cruz Bio-technology, Santa Cruz, CA, USA). The secondary antibody was Goat Anti-Rabbit IgG (Invitrogen). Protein levels were measured by gray value with Quantity One software.

### Statistical analysis

All statistical analyses were performed using the SPSS 17.0 software package (SPSS, Chicago, IL, USA). The significance of differences between groups was estimated by Student's *t*-test and chi-square test. The results are reported as the means ± SDs. Statistical significance was assigned at *P* < 0.05 (*) or *P* < 0.01 (**). All experiments were performed at least three times with triplicate samples.
